# Hesperetin and Capecitabine Abate 1,2 Dimethylhydrazine-Induced Colon Carcinogenesis in Wistar Rats via Suppressing Oxidative Stress and Enhancing Antioxidant, Anti-Inflammatory and Apoptotic Actions

**DOI:** 10.3390/life13040984

**Published:** 2023-04-11

**Authors:** Asmaa K. Hassan, Asmaa M. El-Kalaawy, Sanaa M. Abd El-Twab, Mohamed A. Alblihed, Osama M. Ahmed

**Affiliations:** 1 Physiology Division, Zoology Department, Faculty of Science, Beni-Suef University, Beni-Suef 62521, Egypt; 2 Pharmacology Department, Faculty of Medicine, Beni-Suef University, Beni-Suef 62521, Egypt; 3 Department of Microbiology, College of Medicine, Taif University, Taif 21944, Saudi Arabia

**Keywords:** colon carcinogenesis, 1,2 dimethylhydrazine, hesperetin, capecitabine

## Abstract

Colon cancer is a major cause of cancer-related death, with significantly increasing rates of incidence worldwide. The current study was designed to evaluate the anti-carcinogenic effects of hesperetin (HES) alone and in combination with capecitabine (CAP) on 1,2 dimethylhydrazine (DMH)-induced colon carcinogenesis in Wistar rats. The rats were given DMH at 20 mg/kg body weight (b.w.)/week for 12 weeks and were orally treated with HES (25 mg/kg b.w.) and/or CAP (200 mg/kg b.w.) every other day for 8 weeks. The DMH-administered rats exhibited colon-mucosal hyperplastic polyps, the formation of new glandular units and cancerous epithelial cells. These histological changes were associated with the significant upregulation of colon Ki67 expression and the elevation of the tumor marker, carcinoembryonic antigen (CEA), in the sera. The treatment of the DMH-administered rats with HES and/or CAP prevented these histological cancerous changes concomitantly with the decrease in colon-Ki67 expression and serum-CEA levels. The results also indicated that the treatments with HES and/or CAP showed a significant reduction in the serum levels of lipid peroxides, an elevation in the serum levels of reduced glutathione, and the enhancement of the activities of colon-tissue superoxide dismutase, glutathione reductase and glutathione-S-transferase. Additionally, the results showed an increase in the mRNA expressions of the anti-inflammatory cytokine, IL-4, as well as the proapoptotic protein, p53, in the colon tissues of the DMH-administered rats treated with HES and/or CAP. The TGF-β1 decreased significantly in the DMH-administered rats and this effect was counteracted by the treatments with HES and/or CAP. Based on these findings, it can be suggested that both HES and CAP, singly or in combination, have the potential to exert chemopreventive effects against DMH-induced colon carcinogenesis via the suppression of oxidative stress, the stimulation of the antioxidant defense system, the attenuation of inflammatory effects, the reduction in cell proliferation and the enhancement of apoptosis.

## 1. Introduction

Colorectal cancer (CRC) is the third and second most prevalent cancer in males and females, respectively, around the world. It accounts for 10% of all malignancies and is thought to be the cause of approximately 600,000 deaths each year [1,2]. There are numerous risk factors linked to the development of CRC, including exogenous risk factors such as obesity, lack of physical exercise, nicotine use, moderate-to-excessive alcohol consumption, hypertension, increased blood lipids and colonization by *Streptococcus gallolyticus*, as well as endogenous risk factors, such as a personal or family history of colon polyps and hereditary CRC, inflammatory bowel illness, type 2 diabetes, hereditary nonpolyposis colon cancer (CC) and Cowden’s disease [3,4]. Although CC is frequently discovered in the late stages when the symptoms become clear, the early detection of cancer can save the lives of patients [5].

The most commonly used CC animal model is the 1-dimethylhydrazine (DMH)-induced animal model [6]. Colon tumors produced by DMH, a powerful colon carcinogen, resemble human CC in many ways, including how they react to several promotion- and prevention-related drugs [7]. A number of pathogenic alterations, including the creation of aberrant cryptic foci, occur as a result of DMH-induced CC in a multi-step process [8].

Oxidative stress due to the excessive production of reactive oxygen species (ROS) is a cellular state that overrides the antioxidant defense mechanisms of cells. Many studies have demonstrated a substantial correlation between oxidative stress and the development or advancement of a number of human diseases, including cancer [9,10,11]. Chronic oxidative stress has been connected to cancer in epidemiological studies [12], proving its role in the development of cancer. The function of ROS in tumor genesis, development and progression is supported by a large body of experimental evidence [13,14,15]. Reactive oxygen species are created during typical cellular metabolism. Although ROS generation is essential for healthy cell-signaling pathways, excessive ROS can harm mitochondrial and genomic deoxyribonucleic acid (DNA), causing mutations in molecules, as well as DNA damage [16].

Apoptosis is a tightly controlled physiological process of cell death that eliminates unneeded, severely damaged, mutant, ageing and/or unrepairable cells while maintaining the integrity of the remaining cells and the organism as a whole [17,18]. Apoptosis imbalance, which can involve levels of apoptosis that are either excessively high or excessively low, may contribute to the pathogenesis of a variety of illnesses, including cancer, ischemia, neurodegeneration and autoimmunity [19]. Apoptosis is triggered by toxic carcinogens or mutagenic substances, viral infections and UV light. Extracellular or intracellular cues can commit cells to undergoing apoptosis, which involves activating the caspase family through intrinsic and extrinsic mechanisms [20,21].

The versatile cytokine known as transforming growth factor-beta (TGF-β) was shown to have both physiological and pathological uses. The three main TGF-family isoforms, TGF-β1, TGF-β2 and TGF-β3, exhibit various biological functions [22,23]. Interestingly, only the promoter region of TGF-β1 can be activated directly by reactive oxygen species (ROS), involving different trans-activating proteins, such as plasmin; due to its multiple regulatory sites [24], this highlights its pleiotropic nature in carcinogenesis, fibrogenesis, immunomodulation, cell proliferation and cell differentiation [25,26].

Many biological processes, including death, differentiation and proliferation, have been intensively examined in relation to the molecular pathways of TGF-β signaling [27].

Particularly in cancer, TGF-βsignaling results in many downstream effects in a context-dependent manner. It has two functions: one as a tumor suppressor in pre-malignant cells and the other as a tumor promoter in cancer cells [28]. Through acquired mutations, cancer cells are able to deactivate the tumor-suppressive elements of TGF-β/Smad signaling, whilst tumor-suppressive effects can selectively apply pressure on pre-malignant cells [29].

Surgery and chemotherapy are the key components of the current clinical treatment for CC. Nonetheless, finding new and more potent medications for the treatment of CC is urgently needed due to the development of side effects and the emergence of drug resistance [30].

Capecitabine (CAP) is a fluoropyrimidine-based chemotherapeutic drug used to treat a variety of malignancies, including colon, colorectal and breast cancer [31]. As an antimetabolite, it causes cell-cycle arrest and apoptosis by blocking DNA polymerase. It has estrogenic properties, cytotoxicity, toxicity and teratogenic properties [32,33]. Additionally, CAP and its metabolites have inter-individual variability in their pharmacokinetic characteristics; this is most likely due to variations in the activity of enzymes involved in CAP metabolism [34,35]. Cytotoxic medications not only kill cancer cells but also harm healthy cells. This toxic reaction has led to concern regarding drug dose and has become a factor influencing patients’ quality of life. Bone-marrow suppression, gastrointestinal problems and hair loss are among the most prevalent adverse effects [36].

*Citrus aurantium* L. (Rutaceae) fruit peel contains a naturally occurring flavone called hesperetin (HES), a phytoestrogen with anti-tumor effects [37]. Hesperetin has been shown to apply a cytotoxic mechanism against a variety of cancer cells, including those from breast cancer [38], pancreatic cancer [39], prostate cancer [40], glioblastoma [41], liver cancer [42], kidney cancer [43], colon cancer [44], lung cancer [45], oral cancer [46], esophageal cancer [47], osteosarcoma [48], ovarian cancer [49], thyroid [50], leukemia [51] and others.

The aim of this study was to investigate the potential of the promising effects of HES on colon carcinogenesis, both alone and in combination with CAP, on DMH-induced colon carcinogenesis in rats.

## 2. Materials and Methods

### 2.1. Drugs and Chemicals

The HES (3′,5,7-trihydroxy-4′-methoxy flavanone) and DMH were purchased from Sigma-Aldrich (St. Louis, MO, USA) and stored at 2–4 °C. The CAP was obtained from the Roche Company and stored at 20–25 °C. Carcinoembryonic antigen (CEA)-enzyme-linked immunosorbent assay (ELISA) kit was supplied by R&D Systems (Minneapolis, MN, USA). The primary antibody for TGF-β1 was obtained from ABclonal Technology (Wuhan, China). All other chemicals used in the experimental procedures and assays were of analytical grade.

### 2.2. Animals and Treatment

Fifty adult male Wistar rats with body weight (b.w.) of approximately 100 ± 20 g were obtained from the National Research Center, Doki and Giza, Egypt. They were kept under observation for two weeks prior to the experiment to exclude any with infections at the time at which the study began. The chosen animals were housed in polystyrene-well aerated cages at normal atmospheric temperature (25 ± 5 °C) and humidity (55 ± 5%) and under a 12-h light/dark cycle. During the study period, the rats were provided with water and a normal basal diet. All animal procedures were in accordance with the guidelines and recommendations of the Experimental Animal Ethics Committee for Use and Care of Animals, Faculty of Science, Beni-Suef University, Egypt (ethical approval number BSU/FS/2018/17).

**Figure 1:**
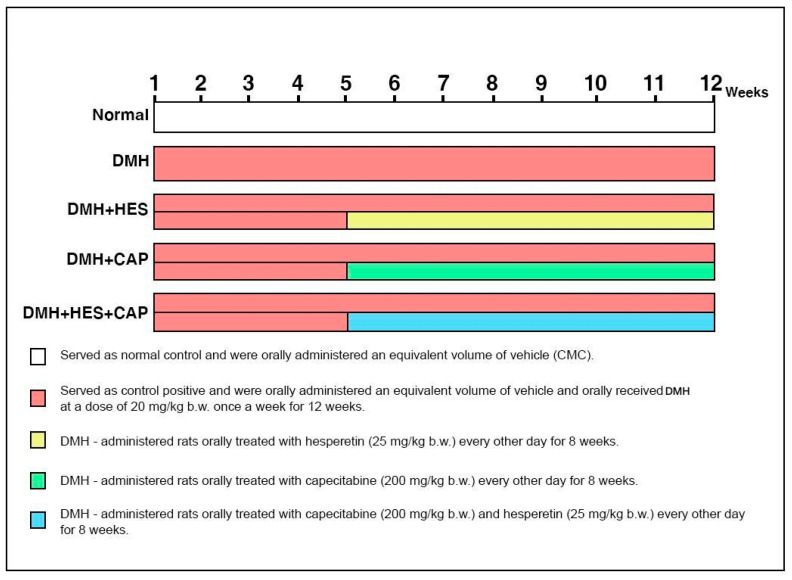
Experimental design.

The experimental animals were randomly allocated into five groups (with ten animals in each), as follows: Group 1 served as a normal control, in which rats were orally administered equivalent volumes of saline (0.9% NaCl) each week for 12 weeks and 1% carboxymethylcellulose (CMC) every other day during the last 8 weeks; the rats in Group 2, the DMH-administered group, were orally given DMH (20 mg/kg b.w.) [52] dissolved in saline (0.9% NaCl) each week for 12 weeks and the equivalent volume of 1% CMC every other day during the last 8 weeks; the rats in Group 3, the DMH-administrated group treated with HES, were orally given DMH as described for Group 2 and orally treated with HES (25 mg/kg b.w.) [53] dissolved in 0.1% CMC every other day for 8 weeks, starting from the 5th week of the DMH administration; the rats in Group 4, the DMH-administered group treated with CAP, were orally given DMH as described for Group 2 and orally treated with CAP (200 mg/kg b.w.) [54] dissolved in 0.1% CMC every other day for 8 weeks, starting from the 5th week of the DMH administration; and the rats in Group 5, the DMH-administered group treated with HES and CAP combination, were orally given DMH as described for Group 2 and orally treated with HES (25 mg/kg b.w.) and CAP (200 mg/kg b.w.) dissolved in 0.1% CMC every other day for 8 weeks, starting from the 5th week of the DMH administration (Figure 1).

### 2.3. Blood and Colon Sampling

After 12 weeks, the animals were given inhalation anesthesia, blood samples from the jugular vein were taken and colon-tissue samples were removed for biochemical, histological and molecular investigations. The animals were then decapitated and dissected. After allowing the blood samples to clot, the sera were separated using centrifugation at 3000 r.p.m. for 15 min. The obtained sera were collected into sterilized tubes and stored at −30 °C. Half gram of each frozen colon was homogenized in 10 mL 0.9% NaCl to yield 1% homogenate (*w*/*v*), and then centrifuged at 3000 r.p.m. for 15 min at 4 °C; the supernatant was separated and kept at −30 °C until it was used for the determinations of oxidative-stress and antioxidant-defense parameters. Other pieces from the colon of each rat were gathered on 10% neutral buffered formalin for histological evaluation and others were stored at −70 °C in sterilized Eppendorf tubes for RNA isolation and real-time PCR (RT-PCR) analysis.

### 2.4. Biochemical Investigations

The serum levels of CEA were estimated using ELISA kits (R&D Systems, Minneapolis, MN, USA), as per the manufacturer’s instructions.

Serum levels of lipid peroxides (LPO) were estimated according to the method described by Preuss et al. [55]. In brief, the proteins were precipitated by adding 0.15 mL 76% trichloroacetic acid (TCA) to 1 mL serum. In order to develop the color of the isolated supernatant, 0.35 mL of thiobarbituric acid (TBA) was added. After 30 min of incubation in an 80 °C water bath, a faint pink color developed and was detected at 532 nm. Malondialdehyde (MDA; 1,1,3,3-tetramethoxypropane) was used as standard. Serum level of reduced glutathione (GSH) content was estimated according to the method described by Beutler [56] by adding 0.5 mL 5,5′-dithiobis(2-nitrobenzoic acid), known as Ellman’s reagent (a color-developing agent) and phosphate-buffer solution (pH 7) to the serum after protein precipitation. The yellow color developed in samples and GSH standard was measured at 412 nm against blank.

The activities of glutathione reductase (GR), glutathione-S-transferase (GST) and superoxide dismutase (SOD) were determined in colon homogenates using the methods presented by Goldberg [57], Mannervik and Guthenberg [58] and Marklund and Marklund [59], respectively. The colon-GR activity was determined by mixing 40 µL of colon-homogenate supernatant with 1 mL substrate (2.2 mmol/L oxidized glutathione) dissolved in buffer (250 mmol/L potassium phosphate; pH 7.3). A volume of 200 µL 0.17 mmol/L NADPH (nicotinamide adenine dinucleotide reduced form) was added and the mixture was incubated in an incubator at 37 °C. The GR activity was calculated from the formula: activity (U/L) = 4983 × ΔA nm/min. To determine colon GST activity, 250 μL mM 1-chloro-2,4-dinitrobenzene (CDNB) was added to a Wasserman tube that contained 250 μL sample, 250 μL GSH solution (4 mM) and 250 μL phosphate buffer (pH 7.3). The developed color was measured after 10 min of incubation at 25 °C at 430 nm. Colon-SOD activity was determined based on the inhibition of auto-oxidation of pyrogallol by the enzyme. The process was dependent on the presence of superoxide ions. The amount of enzyme that caused a 50% inhibition in the extinction changes in 1 min compared to the control was regarded as one unit of the enzyme. Briefly, 50 μL of pyrogallol (10 mM) was added to 1 mL of the colon-homogenate supernatant in the presence of Tris buffer (pH 8). The initial absorbance was measured after adding pyrogallol and at 10 min. The inhibition of the yellow color at 430 nm and the enzyme activity were calculated.

### 2.5. Ribonucleic Acid (RNA) Isolation and Reverse Transcriptase–Polymerase Chain Reaction (RT-PCR) Analysis

The total RNA was separated from the colon tissues based on the method described by Chomczynski and Sacchi [60], using a Qiagen tissue-extraction kit (USA). The isolated RNA was quantified at 260 nm and transcribed into cDNA using My Taq One-Step RT-PCR Kit (Bioline, Meridian Bioscience, Memphis, TN, USA) in the presence of specific primers (LGC Biosearch Technologies, Petaluma, CA, USA) of proliferator marker (Ki67), interleukin-14 (IL-4), proapoptotic protein 53 (p53) and β-actin (Table 1). The resultant PCR products were analyzed following electrophoresis in 1× Tris-Borate-EDTA buffer (pH 8.3–8.5) on 1.5% agarose gel stained with ethidium bromide. A gel-documentation system was used to visualize the electrophoretic pattern. The relative values of gene expression were normalized to that of β-actin.

### 2.6. Histopathological Studies

Colon pieces of each rat were fixed in 10% neutral buffered formalin for 24 h before dehydration in an ascending series of alcohol concentrations, clearing in xylene and embedding in paraffin wax. The paraffin-wax blocks with the tissues were prepared by cutting 5 μm sections. Next, the tissue sections were processed for staining using hematoxylin and eosin (H & E) [64] and the examination was conducted using an electric-light microscope.

### 2.7. Immunohistochemistry

For the immunohistochemical investigations, colon sections (4 µm thick) were mounted onto positive-charged slides (Thermo Fisher Scientific, Pittsburgh, PA, USA) and immunostaining was conducted according to the methods described by Ahmed and Ahmed [65]. Briefly, the sections were incubated in 3% H_2_O_2_ solution for 15 min following deparaffinization, rehydration, antigen retrieval and sealing. Next, they were blocked and incubated with TGF-β1 antibody (Santa Cruz Biotechnology, Santa Cruz, CA, USA) (1:200 dilution) at 4 °C overnight. After washing with phosphate-buffered saline, the sections with the peroxidase-labeled secondary antibody (1:200 dilution) were incubated for 30 min. The bound antibody complex was visualized by the reaction of 3,3-diaminobenzidine (DAB) substrate and counterstaining with hematoxylin. This method was applied according to the instructions of ABclonal Inc. Company, Wuhan, China. The immunohistochemically stained sections were examined by a light microscope at high power (×400). The positive reaction appeared brown in color. The integrated intensities of the TGF-β1 response were measured using the ImageJ program.

### 2.8. Statistical Analysis

The results were expressed as mean ± standard error (SE), which equals SD/√n (n represents the number of animals). All statistical comparisons were made by one-way ANOVA test followed by Duncan’s method for post hoc analysis using Statistical Package for the Social Sciences (SPSS) version 22 for Windows (New York, NY, USA) [66]. Symbols a, b, c and d were used to indicate significance between groups for each parameter. The means, which had different symbols, were statistically significant at *p* < 0.05.

## 3. Results

### 3.1. Effect of HES and CAP on Serum CEA Level

The oral intake of DMH induced a significant (*p* < 0.05) elevation in the serum levels of CEA when compared to the normal control rats. BY contrast, the treatment of the DMH-administered rats with HES and CAP, both individually and in combination, produced a significant improvement (*p* < 0.05) in the serum levels of CEA in comparison with the DMH-administered control (Table 2); the combinatory effect seemed to be the most potent.

### 3.2. Effect on Oxidative-Stress and Antioxidant-Defense Markers

#### 3.2.1. Effects of HES and CAP on Serum Levels of LPO and GSH

The DMH-administered rats exhibited a significant (*p* < 0.05) increase in their serum LPO levels compared to the normal control rats. The oral supplementation of HES and CAP, both individually and in combination, significantly (*p* < 0.05) and successfully prevented the LPO elevation when compared to the DMH-administered control group (Table 3).

By contrast, the serum level of GSH was significantly (*p* < 0.05) decreased in the DMH-administered rats compared to the normal control rats. The supplementation of HES alone and/or in combination with CAP to the DMH-administered rats significantly (*p* < 0.05) prevented the depletion of the serum GSH level when compared to the DMH-administered control (Table 3).

#### 3.2.2. Effects of HES and CAP on Colon SOD, GR and GST Activities in DMH-Administered Rats

The data presented in Table 4 exhibit a significant (*p* < 0.05) decrease in the colon-homogenate activities of the SOD, GR and GST in the DMH-administered group compared with those of the normal control. By contrast, supplementation with the CAP and HES both alone and in combination prevented the depletion of SOD, GR and GST activities (*p* < 0.05). The effect of HES and CAP in combination on the colon GR and GST activities seemed to be the most potent.

### 3.3. Effects of HES and CAP on the mRNA Expressions of Ki67, IL-4 and p53

**Figure 2:**
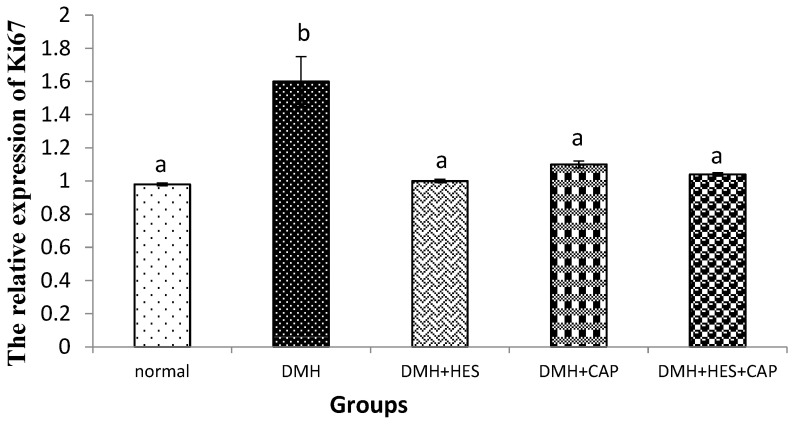
Effects of HES and CAP on Ki76-mRNA expressions in colon tissues of rats given DMH. Data are presented as mean values ± SE with results from 3 independent biological repeats. Means with different symbols (a,b) are significantly different at *p* < 0.05.

The DMH-supplemented rats exhibited a significant (*p* < 0.05) increase in the mRNA expressions of colon Ki67 in comparison with the normal control rats. The treatment with HES alone and in combination with CAP resulted in a significant (*p* < 0.05) decrease in the mRNA expression of ki67 (Figure 2); the effects in the three treated groups were more or less similar.

**Figure 3:**
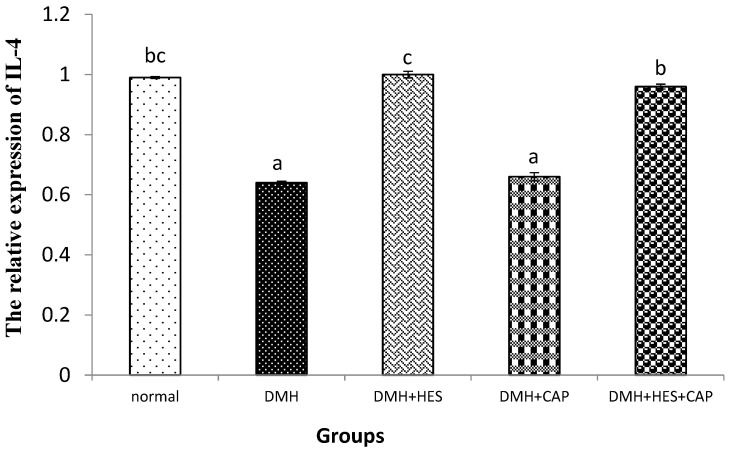
Effects of HES and CAP on IL-4 (B)-mRNA expression in colon tissues of rats given DMH. Data are presented as mean values ± SE with results from 3 independent biological repeats. Means with different symbols (a–c) are significantly different at *p* < 0.05.

As illustrated in Figure 3, the administration of DMH significantly (*p* < 0.05) downregulated the mRNA expression of IL-4 in comparison with the normal control rats. By contrast, the treatment with HES alone and in combination with CAP suppressed the expression (*p* < 0.05) of IL-4, but the effect was not significant (*p* > 0.05) with CAP alone when compared with the DMH-administered group.

**Figure 4:**
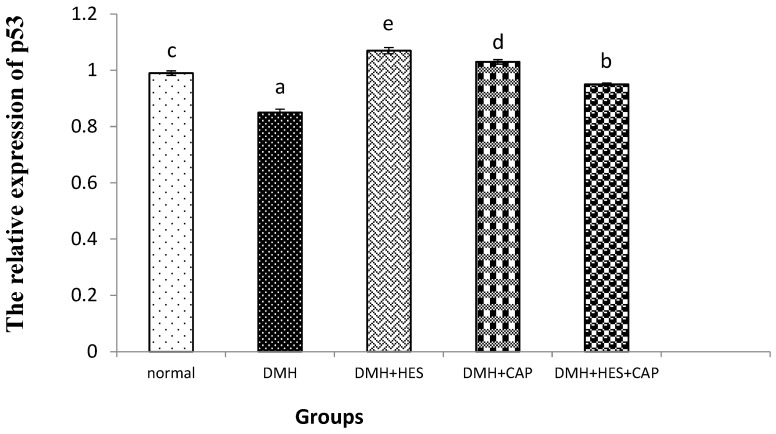
Effects of HES and CAP on p53-mRNA expression in colon tissues of rats given DMH. Data are presented as mean values ± SE with results from 3 independent biological repeats. Means with different symbols (a–e) are significantly different at *p* < 0.05.

The colon-p53-mRNA expression was significantly downregulated in the DMH-administered rats. The treatment of the DMH-administered rats with HES alone and in combination with CAP significantly (*p* < 0.05) suppressed the p53 mRNA expression; the effect of HES seemed to be the most potent (Figure 4).

### 3.4. Histopathological Changes

**Figure 5:**
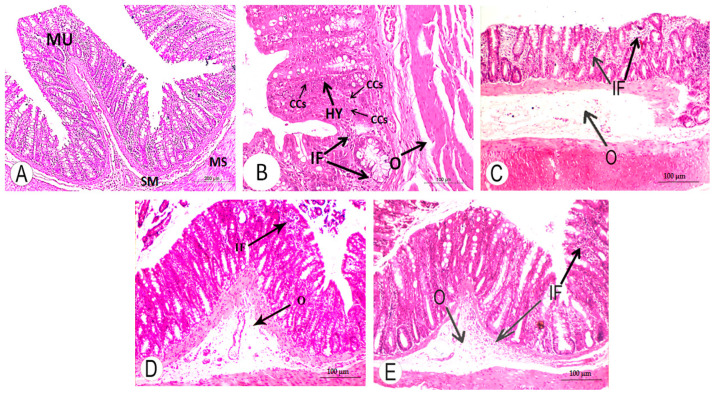
Colon-section photomicrographs of DMH-administered rats treated with HES and CAP, displaying marked improvement in colon architecture. (**A**) Normal control group (CMC), in which the colon has digestive tube with typical histological structures: mucosa (MU), submucosa (SM), muscularis (MS) and serosa/adventitia. (**B**) DMH-administered group, in which the mucosae showed proliferation into the surface epithelial cells (hyperplasia, HY) and cancerous epithelial cells (CCs). Submucosa showed oedema (O). Inflammatory cells (IF) were also observed (H & E × 100). (**C**–**E**): DMH-administered groups treated with HES (**C**), CAP (**D**) and their mixture (**E**) showed focal mucosal inflammatory cell (IF) infiltration and submucosal O (H & E × 100).

The histological architectures of the colon from the normal rats, DMH-administered rats and DMH-administered rats treated with HES and CAP alone or in combination are shown in Figure 5. The colon sections of the normal control rats showed normal histological architectures with typical histological structures of the digestive tube, including the mucosa, submucosa, muscularis and serosa/adventitia (Figure 5A). The colons of the DMH-administered rats (Figure 5B) exhibited changes, such as hyperactivation of the mucosal glands and hyperplastic polyps, hyperplastic activity of the mucosal glands and the formation of new glandular units, hyperplasia of the epithelial cells and cancerous epithelial cells. The submucosa showed oedema. These alterations were amended in the DMH-administered group treated with HES (Figure 5C), CAP (Figure 5D) and their combination (Figure 5E). The colons of these groups exhibited focal mucosal inflammatory-cell infiltration and submucosal oedema. Submucosal inflammatory-cell infiltration was also observed, as shown in Figure 5E.

### 3.5. Effects of HES and CAP on Immunohistochemically Detected TGF-β1

**Figure 6:**
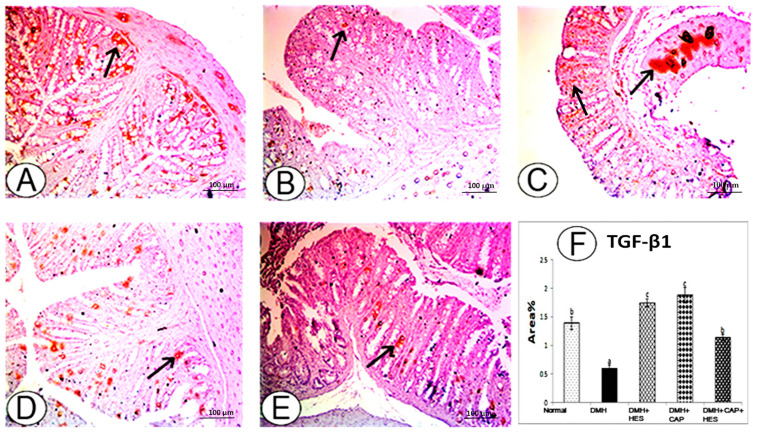
Photomicrographs of rat-colon sections showing the immunohistochemical staining of TGF-β1 in different groups. (**A**) Normal group, showing moderate immunohistochemical staining of TGF-β1 (↑). (**B**) DMH-administered group, showing weak immunohistochemical staining of TGF-β1 (↑). (**C**) DMH-administered group treated with HES, showing strong immunohistochemical staining of TGF-β1 (↑). (**D**) DMH-administered group treated with CAP, showing strong immunohistochemical staining of TGF-β1 (↑). (**E**) DMH-administered group treated with CAP and HES, showing moderate immunohistochemical staining of TGF-β1 (↑). (**F**) Results of image analysis of immunohistochemical staining area percent of TGF-β1 of normal, DMH-administered control and DMH-administered groups treated with HES and/or CAP. Means with different symbols (a–c) are significantly different at *p* < 0.05.

Immunohistochemical staining was used to detect the expressions of the TGF-β1 in the colon tissues of the DMH-administered rats and to evaluate the effects of HES and CAP alone or in combination on DMH-induced colon carcinogenesis. As shown in Figure 6A,B, the colon tissues of the rats in the DMH control group revealed a marked decrease in the number of TGF-β1-positive cells (Figure 6B) compared to the normal controls (Figure 6A). The DMH-administered rats treated with HES and CAP, both individually and in combination (Figure 6C–E), exhibited an increased expression of TGF-β1 when compared to the DMH-administered control.

As depicted in Figure 6F, the DMH control group showed a significant decrease (*p* < 0.05) compared with the normal control group. By contrast, the DMH groups treated with HES and CAP, both individually and in combination, showed strong significant immunohistochemical reactions (*p* < 0.05) of TGF-β1 compared to the DMH control group. The treatments with HES and CAP individually were more potent than the treatment with their combination.

## 4. Discussion

Colorectal cancer is the third most frequent cancer in the world and a leading cause of cancer-related death [67]. Despite the availability of new and innovative medicines, systemic therapy remains the treatment of choice for >25% of patients with metastatic disease [68]. However, the treatment of CRC with chemotherapy results in cytotoxicity and agent resistance [69]. It is thus critical to identify and develop novel compounds with anticancer properties and lower toxicities.

Long-term exposure to DMH has been linked to the development of colon cancer [70]. Azoxymethane (AOM), a metabolite of DMH, is procarcinogen that must undergo metabolic activation in order to produce DNA-reactive byproducts. A reactive metabolite of DMH and AOM called methylazoxymethanol (MAM) rapidly produces the methyldiazonium ion, which can alkylate macromolecules in the liver and colon [71,72,73].

Hesperetin has a long list of pharmacological and biological activities, including antioxidant, anti-cancer, anti-inflammatory and cardiovascular protection [74,75]. Moreover, HES is also known for its significant therapeutic effects and low levels of toxicity for mammals [76,77]. Hence, the present study was conducted to test the effects of HES, both alone and in combination with CAP, for the treatment of colon cancer induced by DMH in rats.

Tumor markers can be utilized as prospective screening techniques and are commonly employed for the early detection of cancer [78]. For example, CEA is a tumor-antigen glycoprotein that is used as a specific index to diagnose people with colon cancer, as patients with advanced cancer conditions have high levels of CEA [79]. The current study found that giving DMH to rats resulted in a significant increase in the serum levels of CEA when compared to control rats. As a strong carcinogen, the DMH caused damage to the colons, followed by instability in colon-cell metabolism, resulting in a number of variations in the levels of CEA, which is a marker of colon function [80]; these results were in agreement with those obtained by Abdel-Hamid et al. [81]. On the other hand, the administration of HES alone or in combination with CAP significantly reduced the serum levels of CEA.

Oxidative stress is caused by an increase in ROS production and a decrease in antioxidant status [82]. It is one of the primary causes of carcinogenesis due to cell harm [83]. Both in vivo and in vitro, the most important process of free-radical production is lipid peroxidation, which has harmful effects on the membrane system and can destroy cells [84]. Lipid peroxidation can cause structural and functional membrane changes, as well as protein oxidation and the production of oxidation products, such acrolein, crotonaldehyde, MDA and 4-hydroxy-2-nonenal (HNE), which are all powerful carcinogens [85,86].

**Figure 7:**
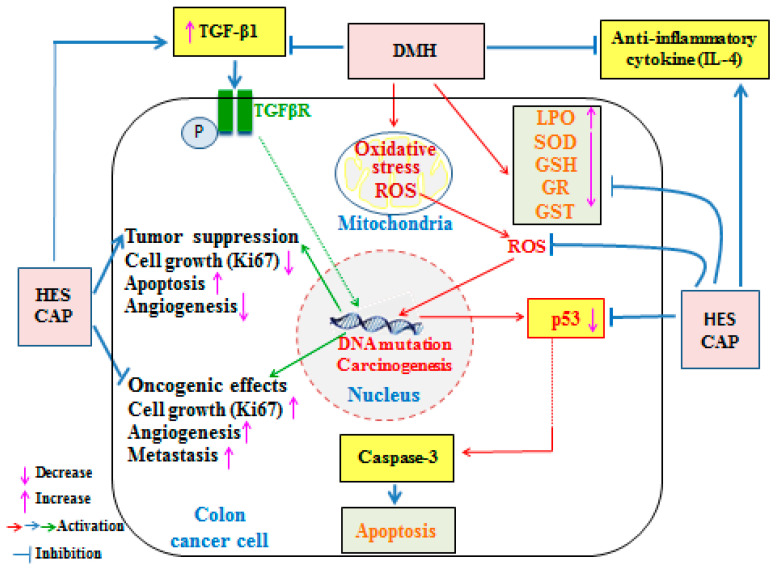
Schematic diagram showing the anticarcinogenic effects of HES and CAP against DMH-induced colon carcinogenenesis via suppression of oxidative stress, inflammation and cell proliferation, as well as induction of cell apoptosis and enhancement of the antioxidant defense system. HES: hesperetin; CAP: capecitabine; DMH: 1,2-dimethylhydrazine; ROS: reactive oxygen species; IL-4: interleukin-4; p53: tumor suppressor protein 53; LPO: lipid peroxides; GSH: glutathione; SOD: superoxide dismutase; GR: glutathione reductase; GST: glutathione-S-transferase; Ki67: proliferator marker.

The flavoprotein oxidoreductase, GR, is responsible for the conversion of oxidized glutathione (GSSG) to its reduced form (GSH), a key component in the ascorbate-glutathione cycle that scavenges H_2_O_2_ [87,88]. Furthermore, GSH is a low-molecular-weight intracellular antioxidant, which serves as a first line of defense. Along with GSH-dependent enzymes such as GST and GR, it detoxifies free radicals produced endogenously, thus performing a crucial protective role [89]. Superoxide dismutase antioxidants are characterized as first-line-defense antioxidants as they act quickly to reduce superoxide radicals [90]. The past findings are in accordance with the results of this investigation, which found that DMH administration resulted in a high serum level of LPO and a low level of serum GSH, in addition to pronounced antioxidant depletion, evidenced by significant decreases in the activities of SOD, GR and GST in colon tissues; this was in contrast to HES administration, either alone or in combination with CAP, due to its antioxidant nature [91,92,93]. The HES also reduced colon oxidative stress, as evidenced by the lower colon MDA levels and higher colon GSH levels. According to Parhiz et al. [94], HES has been proven to have antioxidant properties. It works as an antioxidant in two different ways. The first is direct radical scavenging, which involves neutralizing ROS, such as superoxide anions, hydroxyl radicals and peroxynitrite radicals [95]. The second is an increase in antioxidant-defense biomarkers, such as catalase (CAT), SOD, glutathione peroxidase (GPx), GST and GSH [96,97]. In our study, CAP potentiated the effects of HES on GR and GST-antioxidant-enzyme activities in DMH-administered rats. The improvement in the antioxidant-defense systems in the DMH-administered rats due to the treatment with HES and CAP was associated with the return of the colon histological features to near normal levels with the absence of cancer cells; this led us to suggest that the suppression of oxidative stress and the enhancement of the antioxidant defense system may have an important role in producing the anticarcinogenic effects of HES and CAP in DMH-induced colon carcinogenesis in Wistar rats (Figure 7).

The anti-inflammatory cytokine, IL-4, is emitted by T cells, mast cells, basophils and a subset of natural killer cells [98]. Many functions of activated macrophages are inhibited by IL-4, including the release of reactive oxygen intermediates [99]. It inhibits the synthesis of TNF-α and IL-1 by macrophages [100] and increases the expression of the IL-1 receptor antagonist. It also increases the activity of macrophage 15-lipoxygenase, which may limit the production of the proinflammatory leukotriene B4 [101]. The present investigation showed a significant decrease in the level of IL-4 mRNA due to the DMH administration, while the expression of this interleukin was increased in the rats administered DMH and treated with HES, both alone and in combination with CAP. Thus, both HES and CAP in DMH-administered rats have potent anti-inflammatory actions, in addition to their efficient antioxidant activities (Figure 7).

The above findings were reinforced by the immunohistochemistry analysis of TGF-β1. A multifunctional cytokine, TGF-β1 influences signaling cascades in tumor cells by regulating the entry of inflammatory/immune cells and cancer-associated fibroblasts into the tumor microenvironment. It can inhibit NF-κB activation by interacting with Smad7 [102], inhibiting proinflammatory TNF-α signals as a major modulator of TGF-β1 signaling [103]. The immunohistochemical analysis of TGF-β1 showed this to be evident in this investigation and indicated that HES alone increased the expression of TGF-β1 and, when combined with CAP, restored the expression of TGF-β1 to normal. In gastrointestinal-tumor development and progression, TGF-β signaling has a dual role, acting as both a tumor suppressor and a tumor promoter (Figure 7) in a stage- and context-dependent manner [104,105]. Furthermore, TGF-β signaling functions as a tumor suppressor by encouraging cell-cycle arrest and death during the early stages of tumor development. On the other hand, TGF-β has been demonstrated to enhance tumor-cell proliferation, epithelial–mesenchymal transition and stem-like activity during tumor progression, as well as inflammation and angiogenesis. The transition of TGF-β’s activity from tumor-suppressive to tumor-promoting may be a result of the accumulation of mutations in TGF-β-signaling=pathway components during tumor growth [105]. In the present study, the significant decrease in colon-TGF-β1 expression was associated with a significant increase in the cell-proliferator marker, Ki67 and a decrease in the proapoptotic mediator, p53 in DMH-induced colon cancer. The treatment of the DMH-administered rats with HES and CAP significantly increased the expression of colonic TGF-β1, along with a concomitant decrease in colonic Ki67 and increase in p53. Therefore, TGF-β1 may act as a tumor suppressor under these conditions (Figure 7).

The loss of apoptosis in cancer cells is a critical event in the progression of cancer. Apoptosis is controlled by pro- and anti-apoptotic factor families. Pro-apoptotic (p53 and Bax) and anti-apoptotic genes are involved in cellular growth and apoptosis [106,107]. Cell growth, DNA damage repair and apoptosis are all regulated by the p53 protein [108]. The enhanced malignancy of several major human cancers, including CRC, is associated with an increase in p53 accumulation in the cytoplasm, where the p53 protein is not functional [109]. When compared to normal control rats, those given DMH, in the current study, had colonic cancerous lesions and a significant decrease in the level of colonic p53, which was in agreement with the findings of Gadelmawla et al. [110]. In the present study, the expression of p53 in the colons of the rats given DMH and treated with HES and CAP was high, particularly in the group administered HES. Thus, the induction of apoptosis, as evidenced by the elevated proapoptotic protein, p53, may be involved in the mechanisms of the anticancer actions of HES and CAP (Figure 7).

The proliferation of the cells has been linked to an increased risk of cancer [111]. Furthermore, Ki67 is widely used in pathological investigations to assess cell proliferation in a variety of cancers [112,113,114]. Although Ki67 is expressed at low levels in benign tumors, it is detected at high levels in a variety of malignant lesions and is closely linked to distant metastasis, resulting in a poor patient prognosis. The current investigation found that the Ki67 expression was much higher in the rats given DMH only than in the healthy control rats, which was consistent with the findings of Tong et al. [115]. The treatments used in this study evoked a significant successful lowering of Ki67 expression, preventing additional harm. Thus, the anticancer effects of HES and CAP in the DMH-administered rats may be attributed to their antiproliferative action secondary to the increase in TGF-β1.

## 5. Conclusions

Hesperetin, alone or in combination with CAP, exhibited powerful anti-inflammatory, antioxidant and anti-proliferative effects, as well as the amplification of apoptotic actions, thus preventing DMH-induced colon carcinogenesis. The combinatory effect was the most potent in improving the altered serum CEA levels and colon GR and GST activities in the DMH-administered rats. Nevertheless, with the exception of these effects, HES does not add further potential to the anticarcinogenic effects of CAP. Further studies are required to assess the effects of HES alone or in combination with CAP on human CC xenografts and clinical studies are also required to assess the safety and efficacy of these agents in human beings. An important limitation of this study was its focus on the effect on apoptotic protein p53 only and the lack of measurements of other apoptotic mediators, such as caspase-9 in the intrinsic pathway, caspase-8 in the extrinsic pathway and caspase-3, which is a common mediator in both pathways. Thus, further studies are required to assess the effects on mediators other than p53 to elucidate the full effects on the intrinsic and extrinsic pathways of apoptosis.

## Figures and Tables

**Table 1:** Primer sequences used in qRT-PCR analysis.

Gene	Sequence (5′–3′)	References
**Ki67**	F: 5d CTTTGCGCCATGCTGAAACT3′ R: 5d ATGACGACCTGGAACATCGG3′	Yanai et al. [61]
**IL-4**	F: 5d GGAACACCACGGAGAACG3′ R: 5d GCACGGAGGTACATCACG3′	Zhou et al. [62]
**p53**	F: 5d CAGCGTGATGATGGTAAGGA3′ R: 5d GCGTTGCTCTGATGGTGA3′	Ahmed et al. [63]
**β-actin**	F: 5d TCACCCTGAAGTACCCCATGGAG3′ R: 5d TTGGCCTTGGGGTTCAGGGGG3′	Ahmed et al. [63]

**Table 2:** Effects of HES and CAP on serum CEA levels in DMH-administered rats.

Groups	CEA (ng/mL)
**Normal control**	1.90 ± 0.07 ^a^
**DMH control**	12.83 ± 0.65 ^d^
**DMH + HES**	5.01 ± 0.29 ^bc^
**DMH + CAP**	6.01 ± 0.35 ^c^
**DMH + HES + CAP**	4.17 ± 0.15 ^b^

Data are presented as the mean ± SE (*n* = 6). Means with different superscript symbols (^a–d^) are significantly different at *p* < 0.05.

**Table 3:** Effects of HES and CAP on serum LPO and GSH levels in DMH-administered rats.

Groups	LPO (nmol/mL)	GSH (µmol/L)
**Normal control**	6.04 ± 0.8 ^a^	4.30 ± 0.23 ^d^
**DMH control**	18.10 ± 0.4 ^c^	1.23 ± 0.15 ^a^
**DMH + HES**	13.65 ± 0.62 ^b^	3.83 ± 0.26 ^cd^
**DMH + CAP**	15.00 ± 1.07 ^b^	2.71 ± 0.10 ^b^
**DMH + HES + CAP**	15.55 ±1.15 ^b^	3.69 ± 0.06 ^c^

Data are presented as the mean ± SE (*n* = 6). Within the same column, means with different superscript symbols (^a–d^) are significantly different at *p* < 0.05.

**Table 4:** Effects of HES and CAP on colon SOD, GST and GR activities in DMH-administered rats.

Groups	SOD (U/g)	GR (U/g)	GST (U/g)
**Normal control**	19.8 ± 0.82 ^c^	90.21 ± 4.25 ^b^	632.11 ± 4.71 ^bc^
**DMH control**	3.77 ± 0.21 ^a^	36.38 ± 5.08 ^a^	254.26 ± 28.17 ^a^
**DMH + HES**	10.71 ± 0.24 ^b^	134.54 ± 13.73 ^c^	608.95 ± 10.40 ^b^
**DMH + CAP**	11.44 ± 0.20 ^b^	166.18 ± 13.49 ^cd^	651.09 ± 7.82 ^bc^
**DMH + HES + CAP**	11.46 ± 0.07 ^b^	185.36 ± 15.94 ^d^	658.58 ± 7.24 ^c^

Data are presented as the mean ± SE (*n* = 6). Within the same column, means with different superscript symbols (^a–d^) are significantly different at *p* < 0.05.

## Data Availability

Data are contained within the article.

## Abbreviations

CRC: colorectal cancer; CC: colon cancer; DMH: 1,2-dimethylhydrazine; ROS: reactive oxygen species; DNA: deoxyribonucleic acid; TGF-β: transforming growth factor-beta; CAP: capecitabine; HES: hesperetin; CEA: carcinoembryonic antigen; ELISA: enzyme-linked immunosorbent assay; b.w.: body weight; CMC: carboxymethylcellulose; LPO: lipid peroxides; MDA: malondialdehyde; GSH: reduced glutathione; GR: glutathione reductase; GST: glutathione-S-transferase; SOD: superoxide dismutase; Ki67: proliferator marker; IL-4: interleukin 4, p53: proapoptotic protein 53; RNA: ribonucleic acid; RT-PCR: reverse transcriptase–polymerase chain reaction; MAM: methylazoxymethanol.
